# In Vitro Efficacy of Antivirals and Monoclonal Antibodies against SARS-CoV-2 Omicron Lineages XBB.1.9.1, XBB.1.9.3, XBB.1.5, XBB.1.16, XBB.2.4, BQ.1.1.45, CH.1.1, and CL.1

**DOI:** 10.3390/vaccines11101533

**Published:** 2023-09-28

**Authors:** Andrei A. Pochtovyi, Daria D. Kustova, Andrei E. Siniavin, Inna V. Dolzhikova, Elena V. Shidlovskaya, Olga G. Shpakova, Lyudmila A. Vasilchenko, Arina A. Glavatskaya, Nadezhda A. Kuznetsova, Anna A. Iliukhina, Artem Y. Shelkov, Olesia M. Grinkevich, Andrei G. Komarov, Denis Y. Logunov, Vladimir A. Gushchin, Alexander L. Gintsburg

**Affiliations:** 1 Federal State Budget Institution “National Research Centre for Epidemiology and Microbiology Named after Honorary Academician N. F. Gamaleya” of the Ministry of Health of the Russian Federation, 123098 Moscow, Russia; kustovad70@gmail.com (D.D.K.);; 2 Department of Virology, Biological Faculty, Lomonosov Moscow State University, 119991 Moscow, Russia; 3 Shemyakin-Ovchinnikov Institute of Bioorganic Chemistry, Russian Academy of Sciences, 117997 Moscow, Russia; 4 Moscow Healthcare Department, 127006 Moscow, Russia; 5 Department of Infectiology and Virology, Federal State Autonomous Educational Institution of Higher Education I.M. Sechenov, First Moscow State Medical University of the Ministry of Health of the Russian Federation (Sechenov University), 119435 Moscow, Russia

**Keywords:** SARS-CoV-2, therapies, mAb, antiviral drugs, drug resistance

## Abstract

The spread of COVID-19 continues, expressed by periodic wave-like increases in morbidity and mortality. The reason for the periodic increases in morbidity is the emergence and spread of novel genetic variants of SARS-CoV-2. A decrease in the efficacy of monoclonal antibodies (mAbs) has been reported, especially against Omicron subvariants. There have been reports of a decrease in the efficacy of specific antiviral drugs as a result of mutations in the genes of non-structural proteins. This indicates the urgent need for practical healthcare to constantly monitor pathogen variability and its effect on the efficacy of preventive and therapeutic drugs. As part of this study, we report the results of the continuous monitoring of COVID-19 in Moscow using genetic and virological methods. As a result of this monitoring, we determined the dominant genetic variants and identified the variants that are most widespread, not only in Moscow, but also in other countries. A collection of viruses from more than 500 SARS-CoV-2 isolates has been obtained and characterized. The genetic lines XBB.1.9.1, XBB.1.9.3, XBB.1.5, XBB.1.16, XBB.2.4, BQ.1.1.45, CH.1.1, and CL.1, representing the greatest concern, were identified among the dominant variants. We studied the in vitro efficacy of mAbs Tixagevimab + Cilgavimab (Evusheld), Sotrovimab, Regdanvimab, Casirivimab + Imdevimab (Ronapreve), and Bebtelovimab, as well as the specific antiviral drugs Remdesivir, Molnupiravir, and Nirmatrelvir, against these genetic lines. At the current stage of the COVID-19 pandemic, the use of mAbs developed against early SARS-CoV-2 variants has little prospect. Specific antiviral drugs retain their activity, but further monitoring is needed to assess the risk of their efficacy being reduced and adjust recommendations for their use.

## 1. Introduction

The current global outbreak of disease caused by the SARS-CoV-2 virus, commonly known as COVID-19, has led to more than 770 million confirmed cases and the unfortunate loss of over 7 million lives as of July 2023, according to the World Health Organization (WHO) data [1]. Significant advancements have been achieved in the development and application of preventive and therapeutic techniques to combat the COVID-19 pandemic. mRNA-based vaccines such as BNT162b2(Pfizer) [2], mRNA-1273 (Moderna) [3] and adenovirus-based vaccines (Sputnik V (Gamaleya Research Centre) [4], ChAdOx1 nCoV-19 Vaccine AZD1222 (Oxford, UK, AstraZeneca) [5]) have become widespread, and their efficacy and safety have been demonstrated in clinical trials and real-world practice. In addition, several dozen SARS-CoV-2-specific mAbs, Tixagevimab + Cilgavimab (Evusheld/AZD7442), Sotrovimab (VIR-7831 or GSK-4182136), Regdanvimab (CT-P59), Casirivimab + Imdevimab (Ronapreve/REGN-COV2), and Bebtelovimab (LY-CoV1404 or LY3853113), among others [6,7] and antiviral drugs (Remdesivir, Molnupiravir, and Nirmatrelvir) have been approved and are available in clinical practice.

Contrary to scientists’ expectations in the early stages of the pandemic, the genetic variability of SARS-CoV-2 presents a significant threat to the efficacy of prevention and therapy [8]. Reports emerged in the first year of the practical use of vaccines and monoclonal antibodies indicating a decline in their efficacy related to the spread of new SARS-CoV-2 variants of concern [9,10,11]. Upon the emergence of the Delta variant, the solution to the problem, in terms of vaccination, was the implementation of booster doses. However, this measure did not fully solve the problem. The measures taken significantly slowed down the spread of the infection but did not completely reduce the circulation of the virus. One notable challenge is the ongoing emergence of new variants with stronger immune-escape abilities [9,12]. With the emergence of the SARS-CoV-2 Omicron (B.1.1.529) variant, there are numerous amino acid mutations in its Spike (S) glycoprotein that lead to the unprecedented evasion of neutralizing antibodies [13]. There have been waves of infection caused by different variants at a global scale, even affecting individuals who received multiple doses of COVID-19 vaccines [13].

The virus’ acquisition of mutations, which allows for it to evade monoclonal antibodies, and the decrease in the efficacy of antiviral drugs due to drug-resistance mutations, highlight the need to continuously monitor pathogen variability. It is also important to evaluate the efficacy of preventive and therapeutic strategies against the increasingly prevalent new genetic variants.

We organized a constant monitoring of the virus’ genetic variations in Moscow. This involved collecting data on the variability of SARS-CoV-2, forming an updated collection of isolates, identifying the most common genetic variants, and evaluating the in vitro efficacy of preventative and therapeutic drugs against new virus variants. Based on this information, proposals were formulated to update medical recommendations.

This article presents data on the in vitro efficacy of specific antiviral inhibitors, such as Remdesivir, Molnupiravir, and Nirmatrelvir, as well as monoclonal antibodies including Cilgavimab + Tixagevimab, Sotrovimab, Casirivimab + Imdevimab, Etesevimab, Bamlanivimab, and Regdanvimab, against eight common SARS-CoV-2 genetic lines: XBB.1.9.1, XBB.1.9.3, XBB.1.5, XBB.1.16, XBB.2.4, BQ.1.1.45, CH.1.1, and CL.1.

## 2. Materials and Methods

### 2.1. Sample Collection and RT-PCR Testing

Nasopharyngeal swabs were collected from patients who were positive for SARS-CoV-2. Total RNA was extracted using an “RNA isolation kit to isolate total RNA from animal and bacterial cells, swabs, and viruses on columns” (Catalog number RU-250, Biolabmix, Novosibirsk, Russia). Quantitative reverse transcription PCR was conducted using a SARS-CoV-2 FRT RT-PCR kit (Catalog number EA-128, N.F. Gamaleya NRCEM, Moscow, Russia) according to the manufacturer’s instructions. Specimens with Ct values <30 were selected for whole-genome sequencing.

### 2.2. Cell Culture and Virus Isolation

Vero E6 cell line (ATCC CRL-1586) was maintained in complete Dulbecco’s modified Eagle’s medium (DMEM), supplemented with 10% fetal bovine serum (FBS, HyClone|Cytiva, Logan, UT, USA), 1× GlutaMAX and 1× Antibiotic-Antimycotic solution (all from Gibco, Grand Island, NY, USA). For the isolation of SARS-CoV-2, Vero E6 cells were inoculated with nasopharyngeal swabs, as described in an earlier [14]. When virus-induced cytopathic effects (CPE) were confirmed by visual observation under a microscope, the presence of SARS-CoV-2 was determined by qRT-PCR. Supernatant from the cells was used to determine virus titers (50% tissue culture infectious dose; TCID50/mL) according to the Reed and Muench method [15]. All viral isolation procedures were performed in a biosafety level 3 (BSL-3) laboratory.

### 2.3. Sequencing and NGS Data Analysis

In this work, two whole-genome sequencing technologies (IonTorrent and Oxford Nanopore) were used. The sequencing and data processing for Ion Torrent was previously described [16,17]. In brief, whole-genome amplification of the SARS-CoV-2 virus genome was performed using the ARTIC primers V4 with RT-PCR using BioMaster RT-PCR-Premium (Catalog number RM05-200, Biolabmix, Novosibirsk, Russia). DNA libraries were prepared using the NEBNext Fast DNA Fragmentation and Library Prep Set for Ion Torrent (New England Biolabs, Ipswich, MA, USA) according to the manufacturer’s instructions. DNA sequencing was performed using the Ion 540 Chip and Ion S5XL System (Thermo Fisher Scientific, Waltham, MA, USA). The ARTIC primers were trimmed using Cutadapt v3.1 [18]. Reads were trimmed using a quality filter via vsearch v2.17.0, and reads smaller than 100 nt were discarded [19]. The trimmed reads were aligned with SARS-CoV-2 Wuhan-Hu-1 (MN908947.3) using BWA-MEM v0.7.17-r1188 [20]. Variant calling and consensus sequence generation were performed using FreeBayes v1.3.5 [21], bcftools v1.12 [22], and bedtools v2.30.0 [23]. Regions with less than 10-fold coverage were masked.

A sequencing protocol using Oxford Nanopore technology was performed using Midnight RT PCR Expansion (EXP-MRT001, Oxford Nanopore, Oxford, UK) according to the manufacturer’s instructions. DNA libraries were prepared using a rapid barcoding kit (SQK-RBK110.96, Oxford Nanopore, Oxford, UK) and then run for 24 h on an R9.4.1 flow cell. Basecalling and demultiplexing were performed using the MinKNOW software v.22.10.7. Reads were processed using the ARTIC bioinformatics pipeline [24]. Lineages were assigned with Pangolin v.4.3 using pango-data v.1.21.

### 2.4. Choice of SARS-CoV-2 Variants

The selection of SARS-CoV-2 variants to assess the efficacy of antiviral drugs and monoclonal antibodies was determined by at least one of the following criteria: (1) a significant prevalence of the variant in the genetic landscape, (2) variants that influenced the epidemiological situation in countries worldwide, and (3) endemic variants. Thus, eight variants of the virus were chosen to assess the efficacy of therapeutics. The variants included XBB.1.5.24, XBB.1.16, XBB.1.9.1, XBB.1.9.3, CL.1, CH.1.1, BQ.1.1.45, and XBB.2.9.

### 2.5. Evaluation of the Neutralization Efficacy of Monoclonal Antibody

In this study, we used several monoclonal antibodies, including Cilgavimab + Tixagevimab (Evusheld/AZD7442, AstraZeneca, Cambridge, UK), Sotrovimab (VIR-7831 or GSK-4182136, GSK, Brentford, UK), Casirivimab + Imdevimab (Ronapreve/REGN-COV2, Regeneron, New York, NY, USA and Roche, Basel, Switzerland), Etesevimab (LY-CoV016 or JS016, Eli Lilly and Company, Indianapolis, IN, USA), Bamlanivimab (LY-CoV555 or LY3819253, AbCellera Biologics, Vancouver, British Columbia, Canada and Eli Lilly and Company, Indianapolis, IN, USA), and Regdanvimab (CT-P59, Celltrion, Incheon, Republic of Korea).

Antibody was serially diluted in DMEM with 2% FBS and mixed with 100 TCID_50_ of the SARS-CoV-2. After 1 h incubation at 37 °C, the mixtures were added to Vero E6 cells in a 96-well plate. The CPE was visually assessed after 96h under a microscope. The mAbs were analyzed at a dilution of 1/20. The neutralization titer was determined as the geometric mean of the dilutions from 4 repeats, where a complete reduction in the CPE was detected. The decrease in the mAbs in terms of in vitro efficacy was determined as the ratio of the Wuhan-neutralizing titer to the Omicron-subvariant-neutralizing titer and was expressed in folds.

### 2.6. Evaluation of the Antiviral Efficacy of Drugs

For our research, we chose three antiviral drugs—remdesivir, molnupiravir, and nirmatrelvir—which are used in the treatment of COVID-19. These drugs were approved by both the United States Food and Drug Administration (FDA) [25] and the Ministry of Health of the Russian Federation [26]. Remdesivir and molnupiravir belong to the class of RNA-dependent RNA polymerase inhibitors (RdRp), whereas nirmatrelvir is classified as a 3CLᵖʳᵒ protease inhibitor.

The antiviral activity assay was carried out as described previously [27]. In brief, Vero E6 cells were plated in 96-well plates at a density of 3 × 10^4^ cells per well. After 18 h incubation, different dilutions of the compound in DMEM with 2% FBS were added to the cell monolayer in triplicate and incubated for 1 h at 37 °C. Then, the cells were infected with the corresponding SARS-CoV-2 virus strain at 100 TCID_50_. The virus-induced CPE was evaluated after 72–96 h of infection using an MTT method. The data obtained from the experiment were analyzed using GraphPad Prism 8.0 software. The IC_50_ values, which indicate the concentration of the compound needed to inhibit 50% of the viral cytopathic effects, were determined using nonlinear regression analysis with the log (inhibitor) vs. response equation.

## 3. Results

### 3.1. Characterization of SARS-CoV-2 Genetic Variants in Moscow

**Figure 1:**
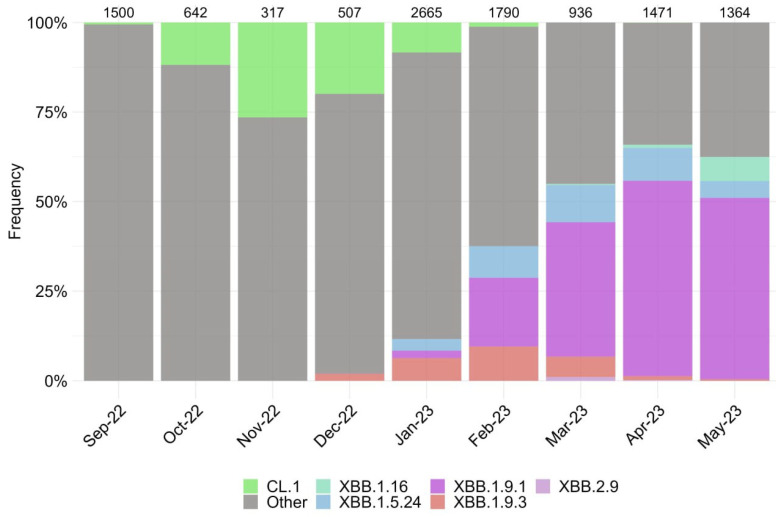
Dynamics of the observed changes in the circulating SARS-CoV-2 variants in Moscow from September 2022 to May 2023. Other lineages are indicated by the color gray. Numerical values displayed above the barplot represent the number of genome-wide sequences that were sequenced within a particular month.

We assessed the changing genetic profile and identified periods of prevalence for specific SARS-CoV-2 variants based on the sequencing of over 11,000 genomes from September 2022 to May 2023 (Figure 1).

The period from September 2022 to January 2023 was characterized by the increase in the endemic CL.1 variant, followed by the emergence of the XBB subvariant and its sublineages, including XBB.1.9* and XBB.1.5.24. During the observation period, the XBB.1.9.1 accounted for the highest proportion, reaching over 55% in April 2023 before its decline. Currently, the prevalence of the XBB.1.16 variant continues to increase. We observed the emergence of two single subvariants, BQ.1.1.45 and CH.1.1, which have not resulted in an increase in new cases in Russia, despite their varying prevalence in other countries worldwide.

According to the data, eight subvariants have garnered significant attention. These subvariants possess a distinctive amino acid changes profile (Figures S1–S3), making them particularly interesting in terms of their potential impact on the efficacy of therapeutics. Consideration of their genetic composition, specifically the receptor-binding domain (RBD) of the S protein, made it possible to identify characteristic amino acid substitutions (Figure S1). Thus, sublineages XBB.1.9.1, XBB.2.9, and XBB.1.5.24 have the same composition of amino acids in the RBD. However, the sublineages of the XBB.1.9.3 and XBB.1.16 variants differed from these by substituting F486S and T478R, instead of F486P and T478K, respectively. Variants BQ.1.1.45 and CL.1 are subline BA.5 and share a similar mutation profile. BQ.1.1.45 differs from XBB variants in the Spike protein at position 339, with aspartic acid residue (D) and valine residue (V) at position 486, indicating the presence of K444T and L452R substitutions, while lacking V445P, G446S, and F490S mutations in the Spike protein (Figure S1). A comparison with BQ.1.1, which was widespread at the end of 2022, revealed that BQ.1.1.45 differs from it only in the S:Y248D (Figure S2) (https://github.com/cov-lineages/pango-designation/issues/1512, accessed on 1 August 2023). Therefore, despite the limited number of cases caused by the BQ.1.1.45 variant, studying it is of practical interest due to the lack of data on the effects of this mutation. The CL.1 variant, compared to BQ.1.1.45, has the K444N substitution (instead of K444T) and does not have R346T. Variant CH.1.1 is a sub-variant of BA.2.75 (B.1.1.529.2.75.3.4.1.1.1.1) and differs from BQ.1.1.45 by not replacing G339D. Instead, it does not replace G339D and contains G446S and F486S. It is important to mention that some of the amino acid profiles we chose were different from those found in other parts of the world. According to GISAID, variants CH.1.1 and BQ.1.1.45 had the S371F substitution in over 90% of cases, but this mutation was not found in our isolated variants (Figure S2). The amino acid composition of several non-structural proteins, specifically nsp5, nsp12, and the ExoN domain of nsp14, was examined to determine their potential impact on the efficacy of tested antiviral drugs. All studied isolates were characterized by the following substitutions: nsp5:P132H, nsp12:P323L, and nsp14:I42V for CH.1.1, and nsp12:G671S for XBB.X. The BQ.1.1.45 variant had unique amino acid mutations, including nsp12:Y273H, XBB.1.16-nsp12:V848I and nsp14:D222Y, CL.1-nsp14:G17R, XBB.1.9.3-nsp14:G44C, and CH.1.1-nsp14:V182I (Figure S3).

### 3.2. Evaluation of Monoclonal Antibody Efficacy In Vitro

We assessed the neutralization activity of mAbs against different Omicron subvariants and the Wuhan-like B.1.1.1 variant. The reduction in neutralizing titers (compared to the Wuhan-like B.1.1.1 virus) for various mAbs against different Omicron subvariants is shown in Table 1.

All studied mAbs exhibited high neutralizing activity against B.1.1.1. All tested monoclonal antibodies, with the exception of sotrovimab (i.e., cilgavimab, ticagevimab, imdevimab, etsevimab, casirivimab, bamlanivimab, and regdanvimab), failed to neutralize the Omicron XBB, BQ, CL and CH subvariants. Sotrovimab showed an 8-, 256-, 304-, and 362-fold reduction against the XBB.1.9.3, XBB.1.5.24, XBB.2.9, and CH.1.1 variants, respectively. These findings indicate that analyzed monoclonal antibodies may be ineffective against the currently circulating virus variants in clinical settings, as previously reported for XBB and BQ.1.1 [28,29].

### 3.3. Evaluation of the Antiviral Efficacy of Drugs In Vitro

**Figure 2:**
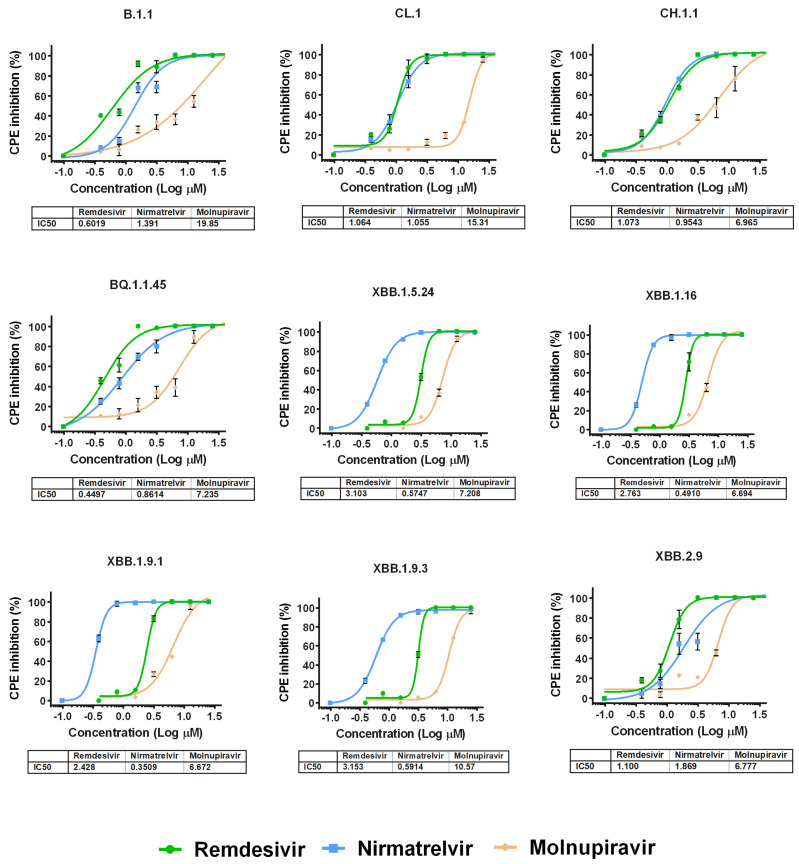
Dose-dependent inhibition curves for antiviral drugs against different SARS-CoV-2 variants. The points represent means ± SEM from triplicate. The tables show IC50 values (µM) for SARS-CoV-2 variants investigated in this study.

We then performed antiviral assays in Vero E6 cells to determine the differences in the suppression of the replication of various Omicron subvariants under the action of remdesivir, nirmatrelvir or molnupiravir. It was found that the IC_50_ values for the studied drugs generally do not differ between variants of SARS-CoV-2, as shown in Figure 2 and Table S1.

Among the studied drugs, molnupiravir had the weakest antiviral effect. For variants XBB.1.5.24, XBB.1.16, XBB.1.9.1 and XBB.1.9.3, remdesivir showed similar activity. For variants B.1.1, CL.1, CH.1.1, BQ.1.1.45 and XBB.2.9, the IC_50_ values for nirmatrelvir and remdesivir were practically the same, but against B.1.1 and XBB.2.9, remdesivir was the most effective. Notably, the IC_50_ values for the molnupiravir in relation to variants B.1.1, CL.1 and XBB.1.9.3 were the highest among the studied variants of SARS-CoV-2 (19.85 µM, 15.31 µM and 10.57 µM, respectively) (Figure S4).

## 4. Discussion

The efficacy of vaccines and therapeutics plays a crucial role in mitigating the morbidity and mortality rates associated with the current phase of COVID-19. In the absence of preventive and therapeutics at the onset of the pandemic, the sole recourse was to implement a system of social distancing measures, such as “lockdown”, which had significant economic, social, and political repercussions [30,31]. Preventing the need for future “lockdowns” is a critical global health objective. However, the ongoing evolution of the SARS-CoV-2 virus in the human population poses certain risks, as new genetic variants of the pathogen can diminish the efficacy of vaccines and therapeutics [8,9,10,11,12]. In the context of the reduced efficacy of drugs, it is imperative to closely monitor the characteristics of the pathogen in order to develop evidence-based healthcare recommendations [32,33]. However, in order to accomplish this objective, monitoring should not be restricted to genetic research. It is imperative to collect viral isolates for subsequent utilization in experimental studies, wherein the efficacy of preventive and therapeutic measures can be consistently evaluated both in vitro and in vivo.

In this study, we presented data regarding the outcomes of the systematic and continuous monitoring of COVID-19 in Moscow. Conducting this monitoring in Moscow, a large metropolis and primary transportation and logistics hub, enables the early detection and isolation of genetic variants of interest. As part of this study, a monthly sequencing of about 2000 samples was carried out, comprising 100 isolates per month. Based on an analysis of the composition of circulating genetic variants and data on morbidity and mortality in Moscow, the Russian Federation, and other countries, we identified eight genetic variants that are of significant interest for further study. The variants XBB.1.9.1, XBB.1.9.3, XBB.1.5, XBB.1.16, XBB.2.4, BQ.1.1.45, CH.1.1, and CL.1 were of the greatest interest. These genetic variants have also been highlighted by other researchers [34,35,36].

Evaluation of the in vitro efficacy of mAb revealed a significant level of neutralizing activity against wild-type SARS-CoV-2 (Wuhan variant). A critical decrease in neutralizing activity against the lines XBB.1.9.1, XBB.1.9.3, XBB.1.16, XBB.1.5, XBB.2.4, and BQ. 1.1.45 (Omicron subvariant) exhibited resistance to the following mAbs: Silgavimab, Tixagevimab, Imdevimab, Etesevimab, Casirivimab, Bamlanivimab, and Regdanvimab. This indicates that a considerable proportion of mAbs exhibit diminished efficacy against the prevailing genetic variants of SARS-CoV-2.

Considering the acquired data, it is necessary to consider the genetic variant of the virus responsible for the specific patient’s illness when utilizing monoclonal antibodies during the present phase of the COVID-19 pandemic. The absence of a genotyping variant system in clinical settings hampers the efficacy of antibody utilization. Thus, our study serves as a valuable addition to the current body of the scientific literature and validates previous observations that the efficacy of mAbs diminishes, rendering these therapies virtually ineffective against Omicron variants [37,38].

Among the mAbs used in our study, Sotrovimab exhibited the most notable efficacy against a range of variants. We attempted to elucidate these findings by considering the available mutations in the examined variants. A notable decrease in the titer of neutralizing antibodies was observed for variant XBB.1.9.1 compared to variant XBB.1.9.3. These variants differ from each other due to a substitution at position 486 of the S-protein, as XBB.1.9.1 has the F486P mutation and XBB.1.9.3 has the F486S mutation. This discrepancy could potentially be attributed to the distinct characteristics of amino acids at position 486. However, it is important to note that the binding epitope of Sotrovimab is located outside the receptor-binding motif (RBM) region [39], specifically within the region encompassing amino acid residues 337–343 of the S-protein [40]. Thus, the substitution at the 486 positions could not, in theory, affect the efficacy of Sotrovimab against XBB.1.9.1 and XBB.1.9.3 variants, so the significant difference that was detected in antibody titers requires further investigation.

In addition, variants XBB.1.5.24 and XBB.2.9 exhibit identical mutations in the RBD to the XBB.1.9.1 variant. However, it is worth noting that the titer reduction factor for these variants was significantly lower, differing by several orders of magnitude. A significant reduction in titer, by 50 thousand times compared to sotrovimab, was observed for BQ.1.1.45 and CL.1 variants. Considering the specific RBD mutation profile exhibited by these variants (Figure S1) and the position of the sotrovimab epitope within the S protein, it is reasonable to hypothesize that Sotrovimab’s inability to neutralize these variants may primarily be attributed to the G339D amino acid substitution. This conclusion is consistent with the findings observed in other variants, as they were characterized by the presence of histidine rather than aspartic acid at position 339 (Figure S2). The reduced efficacy of S309 (sotrovimab precursor) for BQ.1.1, mediated by the presence of mutations G339D and R346T, as these amino acids are involved in interactions with S309, was also shown in an earlier study by Addetia and colleagues [13]. Moreover, similar results for BQ.1.1 with a lack of Sotrovimab activity were demonstrated in other independent studies [28,29]. Therefore, it can be inferred that the application of monoclonal antibodies, which have a fixed composition of active antibodies and do not take into account the specific genetic variant SARS-CoV-2 virus in an infected patient, has limited efficacy at the present stage of the COVID-19 pandemic.

Unlike monoclonal antibodies, the efficacy of all specific antiviral drugs examined in our study was assessed against selected variants of the SARS-CoV-2 virus. Despite the availability of emerging drug-resistance information for certain variants of nirmatrelvir [41,42,43], the specific mutations responsible for conferring this stability have yet to be identified. Despite the increasing knowledge of certain SARS-CoV-2 variants’ resistance to nirmatrelvir, no mutations have been identified as the cause of this resistance. All studied variations contain a solitary P132H substitution in the nsp5 gene (3CLpro), which did not affect the efficacy of antiviral drugs [43]. Therefore, variations in the inhibitory concentration of nirmatrelvir, a specific inhibitor of 3CLpro [44], could potentially be associated with mutations in other genes. The observed variations in the inhibitory concentration of molnupiravir could potentially be associated with additional mutations within the ExoN domain of the non-structural protein nsp14. The exoribonuclease activity 3′-5′ (ExoN) of nsp 14 in SARS-CoV-2 is responsible for the removal of nucleotides that are erroneously incorporated during RNA synthesis by the low-fidelity RdRp enzyme [45]. According to the genome analysis (Figure S3), CL.1 exhibits a substitution at the 17th position of the nsp14 ExoN domain (G17R), while XBB.1.9.3 shows a substitution at the 44th position (G44C). These substitutions may potentially contribute to the higher IC_50_ values observed for molnupiravir, a synthetic ribonucleoside analog N4-hydroxycytidine. Elevated IC_50_ values for remdesivir were observed for XBB.1* variants. Additional sequence analysis identified non-synonymous mutations in the nsp12 gene, which encodes the RNA-dependent RNA polymerase (RdRp). These mutations include: P323L, which was found in all studied variants; G671S, which was present in all variants except BQ.1.1.45 and CL.1; V848I, which was observed in XBB.1.16; Y273H, which was detected in BQ.1.1.45. The significance of these mutations has only been established for P323L, which was found to have no destabilizing effect on RdRp and does not impact resistance [46]. No mutations conferring resistance to remdesivir have been identified [47,48].

The obtained results validate several previous studies regarding the in vitro efficacy of antiviral drugs against various Omicron subvariants [28,49]. Further surveillance of the efficacy of antiviral drugs in response to emerging variants is necessary for the prompt evaluation of the potential decline in their efficacy and to modify guidelines for their administration.

## 5. Conclusions

A timely understanding of the efficacy of prevention and treatment methods is crucial in the fight against COVID-19. Our study demonstrated the efficacy of an integrated approach that includes regular molecular epidemiological monitoring for the rapid detection of new virus variants, using virology to obtain SARS-CoV-2 isolates and evaluate the efficacy of prevention and treatment means, and the development of administrative decisions.

Based on the most recent data, which include the findings of this study, it is apparent that there has been a significant decrease in the efficacy of mAbs Wuhan-like S antigen and the emergence of resistance to antiviral drugs in response to the emergence of new variants of the SARS-CoV-2 virus. This presents a significant challenge for the global community in continuing their efforts in the research and development of novel therapeutic drugs, similar to the modification of the antigenic composition of vaccines against COVID-19.

## Figures and Tables

**Table 1:** Reduction in the neutralization titer (folds) in mAbs against Omicron subvariants in comparison with B.1.1.1 variant (N/D—no data). K—thousands, M—millions.

mAb	XBB.1.9.1	XBB.1.9.3	XBB.1.5.24	XBB.1.16	XBB.2.9	BQ.1.1.45	CL.1	CH.1.1
Cilgavimab	>1.8 M	>1.8 M	>1.8 M	>1.8 M	>1.8 M	>1.8 M	>50 K	>50 K
Tixagevimab	>11.9 M	>11.9 M	>11.9 M	>11.9 M	>11.9 M	>11.9 M	>50 K	>50 K
Sotrovimab	>30 K	8	256	N/D	304	>55 K	362	>30 K
Imdevimab	>2.1 M	>2.1 M	>2.1 M	>2.1 M	>2.1 M	>2.1 M	>50 K	>50 K
Etesevimab	>260 K	>260 K	>260 K	>260 K	>260 K	>260 K	>50 K	>50 K
Casirivimab	>4.2 M	>4.2 M	>4.2 M	>4.2 M	>4.2 M	>4.2 M	>50 K	>50 K
Bamlanivimab	>110 K	>110 K	>110 K	>110 K	>110 K	>110 K	N/D	N/D
Regdanvimab	>8.4 M	>8.4 M	>8.4 M	>8.4 M	>8.4 M	>8.4 M	N/D	N/D

## Data Availability

Viral isolate sequences were deposited in GISAID database: XBB.1.9.1 (Accession ID: EPI_ISL_17480940), XBB.1.9.3 (Accession ID: EPI_ISL_17481024), XBB.1.5.24 (Accession ID: EPI_ISL_17480941), XBB.1.16 (Accession ID: EPI_ISL_17474859), XBB.2.9 (Accession ID: EPI_ISL_17480969), BQ.1.1.45 (Accession ID: EPI_ISL_17480987), CH.1.1 (Accession ID: EPI_ISL_17481102), and CL.1 (Accession ID: EPI_ISL_16053089). Other sequences were deposited in GISAID and VGARus databases (originator: N. F. Gamaleya National Research Center and DCLI Moscow Healthcare Department).

## Supplementary Materials

The following supporting information can be downloaded at: https://www.mdpi.com/article/10.3390/vaccines11101533/s1, Figure S1: Amino acid changes were detected in the receptor-binding domain (RBD) Spike protein of various SARS-CoV-2 variants. These changes were detected: (A) in both the isolates employed in this study and (B) in the viral sequences deposited in the GISAID database. A color gradient is utilized to depict the frequency of changes, where white signifies the absence of occurrences and dark purple signifies 100% occurrence; Figure S2: Amino acid changes were detected in the Spike protein of various SARS-CoV-2 variants. These changes were detected (A) in both the isolates employed in this study and (B) in the viral sequences deposited in the GISAID database. A color gradient is utilized to depict the frequency of changes, where white signifies the absence of occurrences and dark purple signifies 100% occurrence; Figure S3: Amino acid changes were detected in the genome of various SARS-CoV-2 variants. These changes were detected (A) in both the isolates employed in this study and (B) in the viral sequences deposited in the GISAID database. A color gradient is utilized to depict the frequency of changes, where white signifies the absence of occurrences and dark purple signifies 100% occurrence; Figure S4: IC50 values (µM) for SARS-CoV-2 variants investigated in this study. Data are represented as means ± SEM. ANOVA Dunnett’s multiple comparisons test. An asterisk indicates significant differences (*p* < 0.05); Table S1: IC50 values (µM) for SARS-CoV-2 variants investigated in this study.
