# Supplementary material for: In Vitro Efficacy of Antivirals and Monoclonal Antibodies against SARS-CoV-2 Omicron Lineages XBB.1.9.1, XBB.1.9.3, XBB.1.5, XBB.1.16, XBB.2.4, BQ.1.1.45, CH.1.1, and CL.1
Source: Vaccines (Basel). 2023 Sep 28;11(10):1533. doi: 10.3390/vaccines11101533 (PMC10611309; doi:10.3390/vaccines11101533)
Supplement: Supplementary file 1 [file vaccines-11-01533-s001.zip › vaccines-2579664-supplementary.pdf]

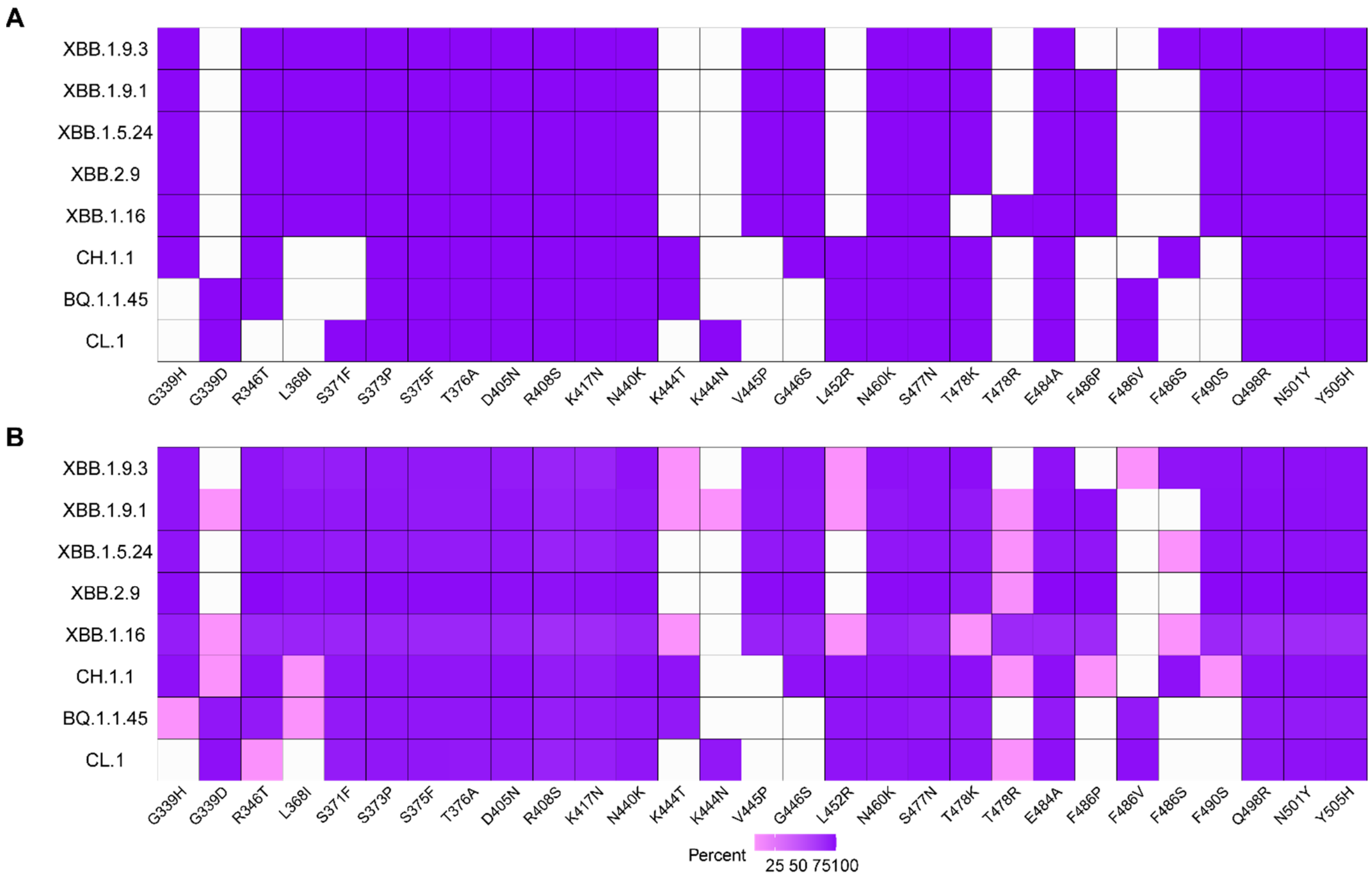

Figure S1: Amino acid changes have been detected in the receptor-binding domain (RBD) Spike protein of various SARS-CoV-2 variants. These changes have been detected (A) in both the isolates employed in this study and (B) the viral sequences deposited in the GISAID database. A color gradient is utilized to depict the frequency of changes, where white signifies the absence of occurrences and dark purple signifies 100% occurrence.

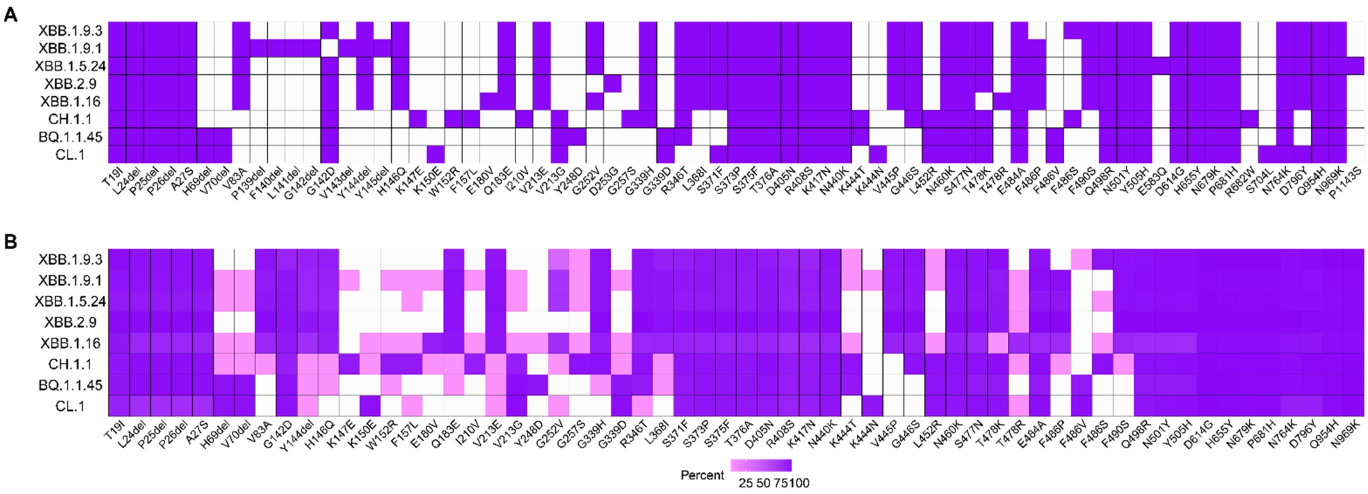

Figure S2: Amino acid changes have been detected in the Spike protein of various SARS-CoV-2 variants. These changes have been detected (A) in both the isolates employed in this study and (B) the viral sequences deposited in the GISAID database. A color gradient is utilized to depict the frequency of changes, where white signifies the absence of occurrences and dark purple signifies 100% occurrence.

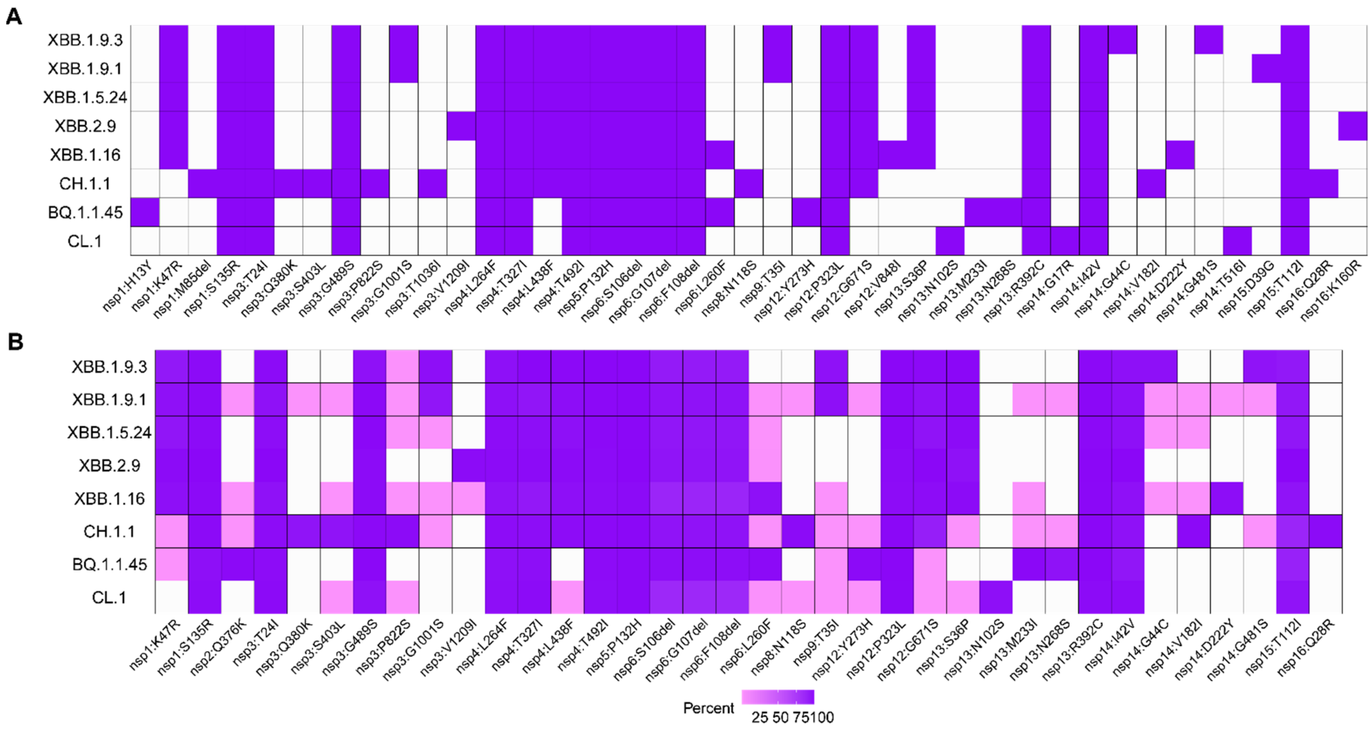

Figure S3: Amino acid changes have been detected in the genome of various SARS-CoV-2 variants. These changes have been detected (A) in both the isolates employed in this study and (B) the viral sequences deposited in the GISAID database. A color gradient is utilized to depict the frequency of changes, where white signifies the absence of occurrences and dark purple signifies 100% occurrence.

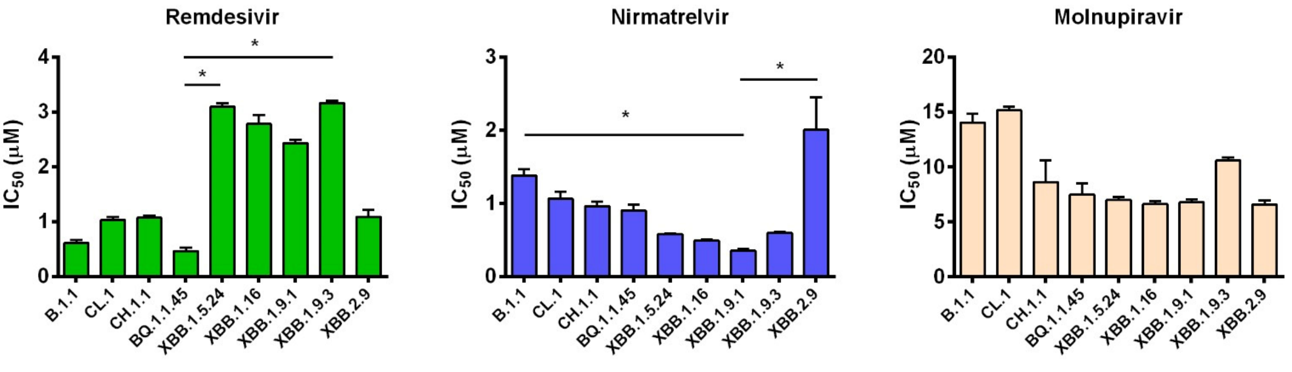

Figure S4. IC50 values (μM) for SARS-CoV-2 variants investigated in this study. Data are represented as means ± SEM. ANOVA Dunnett's multiple comparisons test. An asterisk indicates significant differences ($p < 0.05$).

Table S1. IC50 values (μM) for SARS-CoV-2 variants investigated in this study.

	Remdesivir	Nirmatrelvir	Molnupiravir
B.1.1	0.6019	1.391	19.85
CL.1	1.064	1.055	15.31
CH.1.1	1.073	0.9543	6.965
BQ.1.1.45	0.4497	0.8614	7.235
XBB.1.5.24	3.103	0.5747	7.208
XBB.1.16	2.763	0.491	6.694
XBB.1.9.1	2.428	0.3509	6.672
XBB.1.9.3	3.153	0.5914	10.57
XBB.2.9	1.100	1.869	6.777
